# Functional iron blockade in chronic stress and neurodivergence: a perspective on adaptive stress physiology

**DOI:** 10.3389/fpsyt.2025.1701625

**Published:** 2025-11-03

**Authors:** Simone Hauck

**Affiliations:** ^1^ Graduate Program in Psychiatry and Behavioral Sciences, Universidade Federal do Rio Grande do Sul, Porto Alegre, Brazil; ^2^ Psychodynamic Psychiatry Lab, Hospital de Clínicas de Porto Alegre, Porto Alegre, Brazil

**Keywords:** ferritin, hepcidin, neurodivergence, burnout, stress physiology, HPA axis, ferroptosis, vitamin D

## Abstract

Burnout and trauma are often framed as psychosocial conditions or as dysregulation of the hypothalamic–pituitary–adrenal (HPA) axis. Yet across more than two decades of clinical observation, I have repeatedly encountered a recurring metabolic signature that does not fit existing frameworks: persistent hyperferritinemia without hemochromatosis or overt inflammation, coexisting with low dehydroepiandrosterone-sulfate (DHEA-S) and preserved but gradually declining cortisol dynamics. This constellation is frequently observed in neurodivergent individuals and their families, with early signs already visible in childhood as mild anemia, elevated ferritin, low vitamin D, and behavioral hypervigilance. I propose that this pattern reflects a functional iron blockade (FIB), in which low-grade interleukin-6 signaling upregulates hepcidin, degrades ferroportin, and traps iron intracellularly. While protective against oxidative stress by reducing labile Fe²^+^, the adaptive cost is functional iron deficiency, impaired mitochondrial efficiency, refractory fatigue, and cognitive rigidity. Recognizing this mechanism may refine the understanding of stress-related fatigue and autistic burnout, prevent misdiagnosis as hemochromatosis or incidental hyperferritinemia, and guide research into integrative pathways linking iron metabolism, vitamin D status, and HPA dynamics. This perspective highlights FIB as a potential adaptive but costly response of stress physiology, disproportionately affecting neurodivergent phenotypes.

## Introduction

Burnout and trauma are often discussed as psychosocial conditions or as dysregulation of the hypothalamic–pituitary–adrenal (HPA) axis (1). However, persistent fatigue in these patients often remains unexplained by conventional markers. In long-term clinical practice, I have repeatedly observed a metabolic signature that diverges from existing frameworks: ferritin levels elevated well above expected ranges, in the absence of systemic inflammation, hepatic disease, or genetic hemochromatosis, coexisting with low DHEA-S and preserved morning cortisol.

## Clinical observations

- Adult men: ferritin often 500–700 µg/L+, hemoglobin low-normal, transferrin saturation normal.
- Women of reproductive age: ferritin typically ranges 150–300 µg/L, elevated relative to expected menstrual losses, yet often accompanied by low-normal hemoglobin and hematocrit with normal transferrin saturation—a pattern suggestive of functional iron sequestration rather than true sufficiency.
- Children: early signs include mild anemia, elevated ferritin for age, low 25(OH)D, and behavioral hypervigilance.
- Adults with neurodivergent phenotypes: in some, vitamin D deficiency is persistent despite supplementation, suggesting higher metabolic consumption (2).

These findings are consistent across multiple families, suggesting a trait-like susceptibility. Importantly, they persist despite repeated negative evaluations for C-reactive protein (CRP) and erythrocyte sedimentation rate (ESR), homeostatic iron regulator (HFE) gene mutations, or hepatic disease. They also appear disproportionately frequent in neurodivergent populations—autism spectrum, ADHD, giftedness, and dyslexia—although not exclusive to them (3). Typical laboratory profiles are listed in **Table 1**.

**Table 1:** Clinical profiles suggestive of functional iron blockade in chronic stress.

Feature	Adult men	Women of reproductive age	Children/early expression
Ferritin	500–700 µg/L (very high)	150–300 µg/L (moderate, despite menstrual losses)	>100 µg/L (elevated for age)
Hemoglobin	Normal/low-normal (13–14 g/dL)	Low-normal (12–13 g/dL)	Low-normal (age-adjusted)
Hematocrit	Low-normal	Low-normal	Low-normal
Transferrin saturation	Normal (30%–40%)	Normal (30%–40%)	Normal
Cortisol	Preserved, slowly declining	Preserved, slowly declining	Variable, often preserved
DHEA-S	Consistently low	Consistently low	Low/limitrophe (age-adjusted)
Vitamin D	Variable	Variable	Low 25(OH)D
Inflammatory markers	CRP low	CRP low	CRP low
Symptoms	Fatigue, ruminations, cognitive rigidity	Fatigue, insomnia, poor recovery	Fatigue, hypervigilance, sleep issues
Misinterpretation risk	Mistaken for hemochromatosis	Overlooked as “normal ferritin”	Considered incidental

Summary of typical laboratory findings and symptoms across adult men, women of reproductive age, and children.

## Proposed mechanism

Chronic stress maintains low-grade cytokine signaling (IL-6), upregulating hepcidin. Hepcidin degrades ferroportin, preventing iron export from hepatocytes, enterocytes, and macrophages (4). Iron accumulates as ferritin, whereas serum iron and transferrin saturation remain normal (5, 6). Experimental studies have shown that IL-6 directly induces hepcidin expression through activation of the STAT3 pathway in hepatocytes (7), and this same IL-6/STAT3 axis can be pharmacologically modulated—ferulic acid, for instance, downregulates IL-6 and HAMP expression, reducing hepcidin secretion (8). These findings substantiate the biological plausibility of the proposed mechanism.

- Adaptive function: oxidative protection—reducing circulating Fe²^+^ limits hydroxyl radical formation (Fenton reaction) under chronic stress.
- Clinical cost: functional iron deficiency—impaired mitochondrial efficiency, anemia-like fatigue despite “normal” indices, cognitive rigidity, poor recovery.

**Figure 1:**
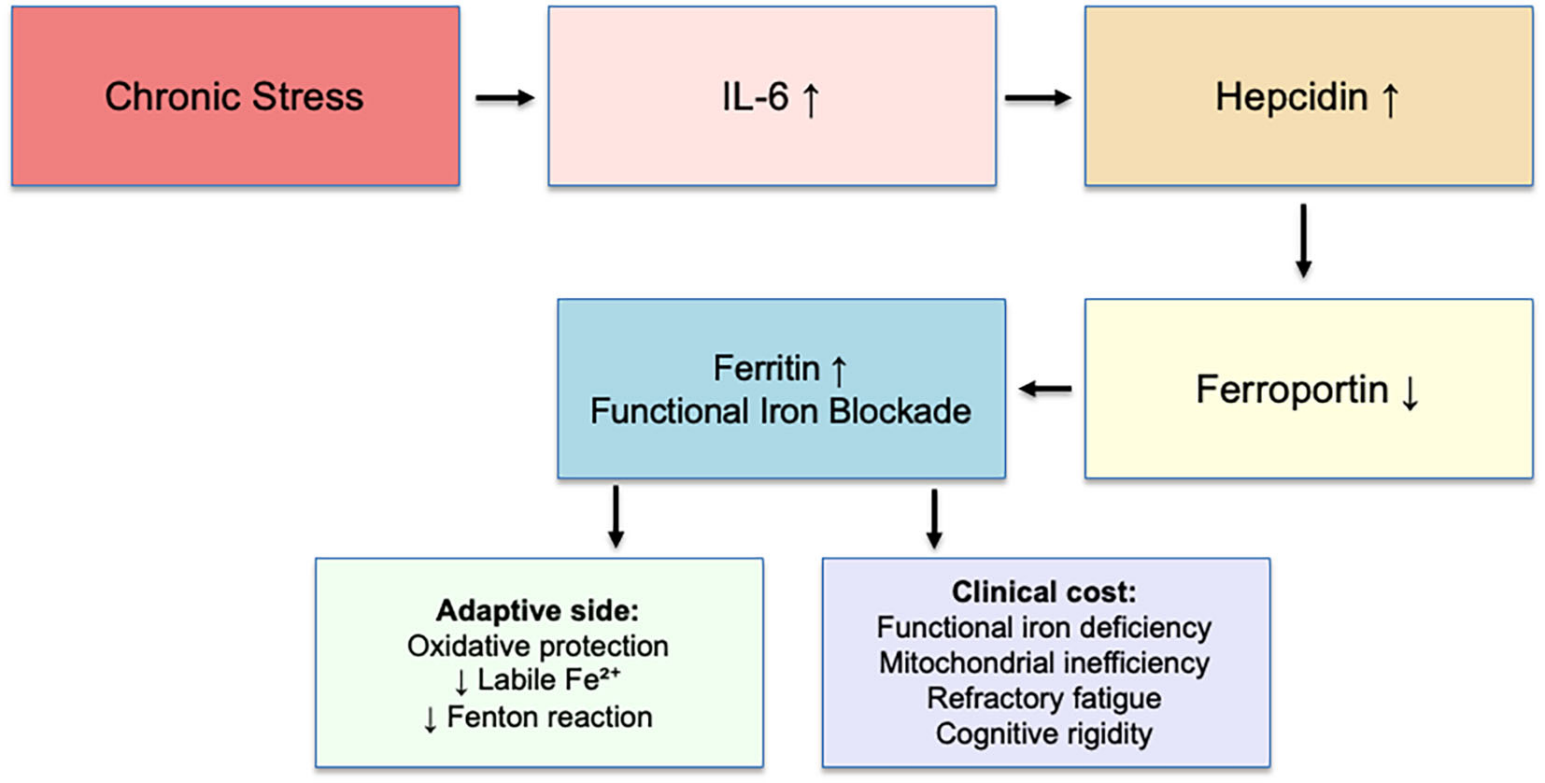
Functional iron blockade mechanism.

Chronic stress maintains low-grade IL-6 signaling, which upregulates hepatic hepcidin. Hepcidin degrades ferroportin, blocking iron export and leading to intracellular iron sequestration and increased ferritin (functional iron blockade, FIB). This adaptive mechanism protects against oxidative stress by reducing labile Fe^2+^ and limiting hydroxyl radical production (Fenton reaction). However, the clinical cost includes functional iron deficiency, mitochondrial inefficiency, refractory fatigue, and cognitive rigidity. This model illustrates FIB as an adaptive yet costly pathway of stress physiology. The adaptive mechanism is summarized in **Figure 1**.

## Preliminary operationalization of FIB

Based on recurring clinical and laboratory constellations, *Functional Iron Blockade* may be provisionally characterized by:

1. Ferritin ≥150 μg/L in women or ≥300 μg/L in men, persisting for ≥3 months in the absence of systemic inflammation (C-reactive protein <3 mg/L, ESR <15 mm/h) or HFE variants causing hemochromatosis;
2. Transferrin saturation within or below the normal range (20%–40%);
3. Low or low-normal hemoglobin/hematocrit relative to ferritin level, suggesting intracellular sequestration rather than true sufficiency;
4. Low DHEA-S (below the age-adjusted 25th percentile) and gradually declining cortisol dynamics despite preserved morning values; and
5. Clinical phenotype of refractory fatigue, cognitive rigidity, and poor stress recovery.

These parameters are intended for exploratory use in research and may evolve with longitudinal validation studies integrating ferritin, hepcidin, HPA hormones, and neurobehavioral outcomes.

## Relationship between FIB and ferroptosis

The IL-6–hepcidin–ferroportin axis central to FIB closely interfaces with ferroptosis, an iron-dependent, lipid-peroxidation-driven form of regulated cell death. Chronic IL-6 signaling increases hepcidin transcription through the STAT3 pathway, promoting ferroportin degradation and intracellular Fe²^+^ retention (7, 8). This adaptive sequestration limits redox-active iron but simultaneously expands the labile iron pool susceptible to Fenton chemistry under oxidative stress (9, 10). Within neurons and other metabolically active cells, this milieu mirrors the pre-ferroptotic state described by Alves et al. (11): Mitochondrial efficiency is downregulated to contain oxidative flux, whereas GPX4-dependent defenses remain functional but strained. In this context, FIB can be interpreted as a sub-threshold, reversible restraint on ferroptotic potential—preserving oxidative safety at the expense of energetic capacity. Persistent IL-6–driven hepcidin expression, if unrelieved, may tilt this adaptive blockade toward maladaptive ferroptosis, as described in chronic stress and neuropsychiatric phenotypes (12).

## Vitamin D–hepcidin axis

Vitamin D not only influences bone and immune homeostasis but also directly regulates iron trafficking by suppressing *hepcidin* transcription through vitamin D receptor (VDR) binding to a response element in the *HAMP* promoter (13). This mechanism rapidly decreases circulating hepcidin, thereby facilitating ferroportin-mediated iron export from monocytes and hepatocytes. Human trials confirm that vitamin D3 supplementation can reduce hepcidin concentrations within 24–48 h (14, 15), and translational studies in monocytes corroborate that VDR activation downregulates *HAMP* expression and restores iron efflux capacity (16). These effects reinforce the role of vitamin D deficiency as a facilitator of chronic intracellular iron sequestration under IL-6–mediated signaling—particularly relevant to neurodivergent profiles, where low 25(OH)D levels are consistently reported (2).

## Implications and discussion

The pattern described here—ferritin elevation without inflammation or genetic iron overload—suggests a distinct adaptive mechanism rather than pathology. The model posits that under chronic stress, mild IL-6 activity increases hepcidin, reducing iron export and promoting intracellular storage. The trade-off protects against oxidative damage but leads to functional iron deficiency and energy inefficiency. The intersection between psychological stress, inflammation, and iron metabolism has been recently emphasized in human studies: Psychological stress activates the HPA axis, elevates IL-6, and induces hepcidin, resulting in functional iron deficiency even with adequate intake (17). This provides further support for interpreting FIB as a psychoneuroendocrine adaptation rather than a static hematologic condition.

Clinical recognition of this pattern may explain refractory fatigue in burnout and NDV profiles where conventional iron indices appear normal. Misdiagnosis remains common, as elevated ferritin can be mistaken for hemochromatosis or incidental finding. Integrating biochemical markers (ferritin, hepcidin, transferrin saturation, DHEA-S, 25(OH)D, and cortisol dynamics) into stress-related research could clarify adaptive versus maladaptive stages of this mechanism.

Elucidating the fine boundary between adaptive regulation and the subsequent failure of these compensatory systems is crucial—not only for advancing research and mechanistic understanding but also for designing individualized clinical interventions. Poorly timed or miscalibrated therapeutic strategies may inadvertently increase oxidative load on an already strained system, collapsing rather than supporting the remaining adaptive capacity. Recognizing this risk is particularly relevant for neurodivergent individuals, whose biological regulation may operate closer to the threshold of energetic collapse. Such consideration aligns with the emerging concept of *autistic burnout*, in which sustained physiological stress responses eventually erode compensatory mechanisms, leading to exhaustion and functional shutdown.

## Future directions

Prospective studies should evaluate FIB-related biomarkers—ferritin, hepcidin, cortisol (including diurnal profile and CAR), DHEA-S, and 25(OH)D—in parallel with neurocognitive and autonomic measures in burnout, trauma, and NDV cohorts. The relationship between IL-6/STAT3 activity, vitamin D status, and ferroptotic vulnerability warrants exploration using integrated endocrine and redox biomarkers. Translational research may clarify how adaptive mechanisms (iron sequestration and mitochondrial downregulation) evolve into clinical exhaustion or neuroinflammatory states. Understanding this adaptive-to-maladaptive continuum may lead to preventive strategies integrating light exposure, nutrition, hormonal modulation, and antioxidant support.

## Conclusion

Functional iron blockade represents an adaptive stress response to chronic overload of the HPA and immune systems, disproportionately affecting neurodivergent phenotypes. Recognizing this mechanism may refine our understanding of refractory fatigue, prevent misdiagnosis, and guide new preventive strategies— linking iron metabolism, vitamin D status, and HPA dynamics into psychoneuroendocrinology research.

## Data Availability

The original contributions presented in the study are included in the article/supplementary material. Further inquiries can be directed to the corresponding author.
